# A novel, yet simple MLC‐based 3D‐crossfire technique for spatially fractionated GRID therapy treatment of deep‐seated bulky tumors

**DOI:** 10.1002/acm2.12826

**Published:** 2020-02-08

**Authors:** Damodar Pokhrel, Matthew Halfman, Lana Sanford, Quan Chen, Mahesh Kudrimoti

**Affiliations:** ^1^ Medical Physics Graduate Program Department of Radiation Medicine University of Kentucky Lexington KY USA

**Keywords:** 3D‐MLC Crossfire, Bulky‐tumors, cerrobend GRID‐block, dose‐escalation

## Abstract

**Purpose:**

Treating deep‐seated bulky tumors with traditional single‐field Cerrobend GRID‐blocks has many limitations such as suboptimal target coverage and excessive skin toxicity. Heavy traditional GRID‐blocks are a concern for patient safety at various gantry‐angles and dosimetric detail is not always available without a GRID template in user’s treatment planning system. Herein, we propose a simple, yet clinically useful multileaf collimator (MLC)‐based three‐dimensional (3D)‐crossfire technique to provide sufficient target coverage, reduce skin dose, and potentially escalate tumor dose to deep‐seated bulky tumors.

**Materials/methods:**

Thirteen patients (multiple sites) who underwent conventional single‐field cerrobend GRID‐block therapy (maximum, 15 Gy in 1 fraction) were re‐planned using an MLC‐based 3D‐crossfire method. Gross tumor volume (GTV) was used to generate a lattice pattern of 10 mm diameter and 20 mm center‐to‐center mimicking conventional GRID‐block using an in‐house MATLAB program. For the same prescription, MLC‐based 3D‐crossfire grid plans were generated using 6‐gantry positions (clockwise) at 60° spacing (210°, 270°, 330°, 30°, 90°, 150°, therefore, each gantry angle associated with a complement angle at 180° apart) with differentially‐weighted 6 or 18 MV beams in Eclipse. For each gantry, standard Millenium120 (Varian) 5 mm MLC leaves were fit to the grid‐pattern with 90° collimator rotation, so that the tunneling dose distribution was achieved. Acuros‐based dose was calculated for heterogeneity corrections. Dosimetric parameters evaluated include: mean GTV dose, GTV dose heterogeneities (peak‐to‐valley dose ratio, PVDR), skin dose and dose to other adjacent critical structures. Additionally, planning time and delivery efficiency was recorded. With 3D‐MLC, dose escalation up to 23 Gy was simulated for all patient's plans.

**Results:**

All 3D‐MLC crossfire GRID plans exhibited excellent target coverage with mean GTV dose of 13.4 ± 0.5 Gy (range: 12.43–14.24 Gy) and mean PVDR of 2.0 ± 0.3 (range: 1.7–2.4). Maximal and dose to 5 cc of skin were 9.7 ± 2.7 Gy (range: 5.4–14.0 Gy) and 6.3 ± 1.8 Gy (range: 4.1–11.1 Gy), on average respectively. Three‐dimensional‐MLC treatment planning time was about an hour or less. Compared to traditional GRID‐block, average beam on time was 20% less, while providing similar overall treatment time. With 3D‐MLC plans, tumor dose can be escalated up to 23 Gy while respecting skin dose tolerances.

**Conclusion:**

The simple MLC‐based 3D‐crossfire GRID‐therapy technique resulted in enhanced target coverage for de‐bulking deep‐seated bulky tumors, reduced skin toxicity and spare adjacent critical structures. This simple MLC‐based approach can be easily adopted by any radiotherapy center. It provides detailed dosimetry and a safe and effective treatment by eliminating the heavy physical GRID‐block and could potentially provide same day treatment. Prospective clinical trial with higher tumor‐dose to bulky deep‐seated tumors is anticipated.

## 1 INTRODUCTION

Spatially fractionated GRID therapy with megavoltage (MV) x‐ray beams has proven to be an effective treatment modality for shrinking bulky (>8 cm, in diameter) malignant tumors.^1^ Traditional GRID therapy treatments have shown great tumor response of bulky lesions with an overall response rate increase of 62% to 91% when they were treated with a single‐dose of GRID therapy (≥15 Gy) followed by conventional extremal beam radiotherapy.^2^ Another study of 71 patients with advanced or bulky tumors of varying histologies demonstrated that 78% response rate for pain palliation and 58.5% and 72.5% objective clinical response rate for mass effect after GRID therapy of 10 to 20 Gy dose with or without additional external beam radiation.^3^ All these early clinical studies demonstrated no significant skin toxicity with GRID therapy.

Currently, MV GRID therapy treatments are delivered using a high attenuation GRID‐block with divergent holes, with step and shoot multileaf collimator (MLC) control points, and/or Tomotherapy machines.^4^, ^5^, ^6^, ^7^, ^8^, ^9^, ^10^, ^11^, ^12^, ^13^, ^14^, ^15^ Although, the treatment planning studies for GRID therapy using tomotherapy and step and shoot MLCs are evolving, these techniques require longer treatment times due to beam modulation and need patient‐specific quality assurance (QA). Moreover, noninterdigitating MLCs potentially may not allow an efficient implementation of this method. The commercial availability of the standard traditional GRID‐block is very limited in each radiotherapy clinic and it is very difficult to design. Additionally, treating deep‐seated bulky‐tumors with traditional single‐field Cerrobend GRID‐blocks could have major limitations such as suboptimal target dose and potentially unwarranted skin toxicity. Heavy traditional GRID‐blocks are a concern for patient safety at various slanted gantry‐angles and dosimetric detail may not be available readily without GRID‐block template in the user's treatment planning system (TPS).

There are several studies suggesting that high doses of radiation (>15 Gy) cause an environment of potential lethal damage making tumor cells more sensitive to further doses of radiation. This is due to the endothelial cells of the tumor microvasculature.^17^, ^18^, ^19^, ^20^, ^21^ Therefore, killing endothelial cells or obstructing small capillaries inside of the tumor will result in an avalanche of tumor cell deaths due to bystander killing in cells adjacent to irradiated regions. The GRID therapy approach takes advantage of this bystander effect that can result in de‐bulking of large tumors.^21^

Our clinical experience is that when using a single‐field GRID‐block, deep‐seated bulky tumors can receive about 1/3 or less of 15 Gy prescription dose, delivering a sub‐optimal treatment to these patients. In this setting, skin toxicity is a major concern when escalating tumor dose. As mentioned above, traditional GRID‐block is not readily available to the radiotherapy clinics and designing and mounting this heavily lifted cerrobend GRID‐block to the Linac head poses a serious concern for patient safety. Therefore, the MLC‐based 3D‐crossfire technique can substantially escalate tumor dose to deep‐seated bulky masses, deliver a more accurate and faster treatment, reduce dose to skin and other critical structures while avoiding patient safety concerns. Herein, we propose and validate a simple, yet clinically useful 3D‐MLC crossfire technique that can be used at any radiotherapy clinic for possible same day treatments of deep‐seated bulky tumors with potentially escalated tumor doses.

## 2 MATERIALS AND METHODS

### 2.1 Patient setup and CT simulation

This retrospective study included 13 patients with deep‐seated bulky lesions. Each patient had different primary diseases and treatment sites as shown in Table 1. Each patient was immobilized using a VacLoc (CIVCO system, Orange City, IA) bag in the supine position. All planning computed tomography (CT) images were acquired on a GE Lightspeed 16 slice CT scanner (General Electric Medical Systems, Waukesha, WI). The 3D‐CT images were acquired with 512 × 512 pixels at 2.5 mm slice thickness and the patients were tattooed/marked. The 3D CT images were then imported into the Varian Eclipse TPS (Varian, Palo Alto, CA). The treating physician delineated the gross tumor volume (GTV) in 3D‐CT images in Eclipse TPS. Mean GTV was 638 ± 455 cc (range: 129 to 1678 cc) with a corresponding tumor diameter of 10.2 ± 2.3 cm (range: 6.0 to 15.0 cm). The site‐specific critical structures were delineated in CT images including skin for dose reporting. The skin contour was generated within 5 mm of the patient body contour. In Eclipse, the skin to tumor center was estimated by using the tumor radius and its proximity to the skin contour. The maximal dose to 2 cm away in any directions from the GTV (D2cm) was calculated for plan evaluation.

**Table 1:** Main tumor characteristics of the patients included in this study.

Pt. #	Treatment site	GTV vol. (cc)	GTV diameter (cm)	Primary disease site
1	Left lung	554	10	Connective and soft tissue of thorax
2	Left neck	129	6	Squamous cell carcinoma of neck
3	Right axilla	503	10	Malignant neoplasm of axilla
4	Left kidney	856	12	Malignant neoplasm of kidney
5	Right neck	512	10	Malignant neoplasm of neck
6	Right kidney	1486	14	Malignant neoplasm of kidney
7	Thyroid	224	8	Malignant neoplasm of thyroid gland
8	Chest	442	9	Squamous cell carcinoma of chest
9	Chest	551	10	Malignant neoplasm of neck/chest
10	Abdomen	467	10	Intra‐abdominal lymph nodes
11	Liver	366	9	Intra‐abdominal lymph nodes
12	Right adrenal	1678	15	Neoplasm of cortex of adrenal gland
13	Right thigh	530	10	Neoplasm of urinary organ

### 2.2 Clinical GRID‐block plans and treatment delivery

All 13 patients listed above were treated using a single‐field Cerrobend GRID‐block in our clinic to a maximum prescription dose of 15 Gy in one fraction. This block was designed by casting a hexagonal array of divergent holes of 14.3 mm diameter with 21.1 mm center‐to‐center spacing projected at the isocenter plane. The apertures were machine milled in a 7.5 cm thick Cerrobend block. The patients were simulated using the source‐to‐surface distance (SSD) technique, with SSD = 100.0 cm. The monitor unit (MU) settings for single‐field GRID‐blocks were calculated by using a look up table as follows: MU = Prescribed dose/[cGy/MU (d_max_) × %DD (r, d)], where r is the GRID‐field size and d is the tumor depth. A detailed description of the clinical implementation and validation of this GRID‐block can be found in Meigooni et al.^22^ 6 or 18 MV photon beams were used based on tumor depth on a Clinac 21EX machine. A dose rate of 400 MU/min was used per commissioning data. With this GRID‐block, various field sizes up to a maximum of 25 × 25 cm can be achieved. On a per‐patient basis, depending on the tumor‐size, part of the radiation field can be blocked by using tertiary MLCs in the Linac head. However, it would be difficult to accurately estimate the skin dose due to the absence of GRID‐block template in Eclipse TPS.

On the treatment day, these patients were setup using 100 cm SSD followed by a verification port film with GRID‐block setup before treatment. The treatment was delivered once the GRID‐block setup was verified by the treating physician.

### 2.3 3D‐MLC crossfire plans

**Figure 1:**
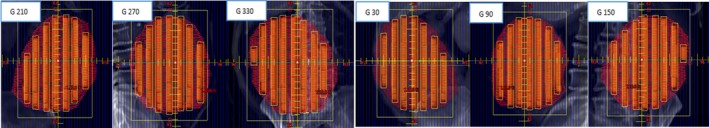
Demonstration of the three‐dimensional‐multileaf collimator (3D‐MLC) fit to the grid pattern for each gantry angle used (example case #12, right adrenal). The original gross tumor volume (GTV) contour is shown in red with orange showing the grid‐pattern generated within the original GTV contour for MLC‐based 3D‐crossfire planning.

All 13 patients (multiple primary disease sites, see Table 1) who underwent conventional single‐field GRID‐block therapy as described above were re‐planned using a simple MLC‐based 3D‐crossfire method. For each patient, a GTV contour was used to generate a 10 mm diameter and 20 mm center‐to‐center distance grid‐pattern mimicking the conventional GRID‐block using an in‐house MATLAB program. The program read the 3D‐CT images and structure set (GTV contour) in DICOM format. A voxel mask of the grid lattice structure was created inside the GTV structure using MATLAB’s boundaries function in DICOM format. The lattice structure was then imported into Eclipse for 3D‐MLC based crossfire planning. The 3D‐MLC crossfire GRID plans were generated using source to axis distance (SAD) method by choosing an isocenter at the tumor center. In our cross‐firing technique, 6 co‐planar gantry angles (210°, 270°, 330°, 30°, 90°, and 150°) were used with differentially weighted 6 or 18 MV photon beams. All gantry angles had a 90° collimator rotation achieving a GRID shaped tunneling dose distribution. All head and neck and chest tumors used 6 MV beams and abdominal and pelvis plans were calculated with 18 MV beams. Standard Millenium120 (Varian Medical System, Palo Alto, CA) 5 mm MLC leaves were fit to the grid‐lattice with a 90° collimator rotation for all gantry angles used. Overall, planning time was about an hour or less for an experienced physicist. Figure 1 shows the beam‐eye views (BEV) of the GRID structure pattern fitted with MLC for each gantry angle.

For a similar prescription of 15 Gy in one fraction, the dose rates of both 400 MU/min (similar to traditional cerrobend GRID‐block) and 600 MU/min were tested. An advanced Acuros‐based dose calculation algorithm with 2.5 mm calculation grid size was used for heterogeneity corrections.^23^, ^24^

### 2.4 Independent dose verification

For physics 2nd check, for these 3D‐MLC plans, field‐by‐field MU calculations were verified independently by using the most commonly used tissue to maximum ratio (TMR) based in‐house spreadsheet calculation. Additionally, these plans were re‐calculated using an in‐house Monte Carlo (MC) program^28^, ^29^ based on PENELOPE MC code^30^ by utilizing a vendor provided phase space file. MLCs were modeled following the schematic drawing provided by the vendor. A detailed description of the clinical implementation and validation of our in‐house MC algorithm can be found in the literature cited above.

### 2.5 3D‐MLC patient setup and treatment delivery

These patients can be initially positioned using external marks and in‐room lasers followed by anterior‐posterior and lateral kilovoltage (kV) or MV image pairs for set up verification. If desired, soft tissue alignment can be achieved by acquiring a kV conebeam CT scan before treatment delivery. Per planning approach, adopting a continuous clockwise gantry angles timing could potentially deliver a faster and more effective 3D‐MLC grid treatment.

### 2.6 Plan evaluation

For the target dose, the parameters evaluated include mean dose to GTV, GTV dose heterogeneities and the peak‐to‐valley dose ratio (PVDR). The indication of the dose heterogeneities is usually measured by the PVDR and is defined as the ratio of maximum to minimum dose inside the target. The maximum dose is located under the MLC open area and minimum dose is located under the MLC‐block shielded area. The site‐specific dose to the organs at risk (OAR) were evaluated including the skin dose for all cases. The lung SBRT RTOG‐0915 protocol with 34 Gy single‐fraction (Arm 1) dose tolerances were adopted for the OAR dose evaluation.^25^ Dose limits for maximum doses to spinal cord < 14.0 Gy, heart < 22.0 Gy, esophagus < 15.4 Gy, maximum dose and dose to 1 cc of ribs, <30.0 and <22.0 Gy, maximum dose and dose to 10 cc of skin <26.0 and <23.0 Gy; maximum dose and dose to 10 cc of stomach and bowel <12.4.0 and <11.2 Gy, respectively were used similar to single‐dose lung SBRT protocol recommendations.

Additionally, the total number of MU and beam on times were recorded. Statistical analysis was performed using Microsoft Excel (Microsoft Corp., Redmond, WA) data analysis program. Two‐sided paired student’s *t*‐test was used to evaluate parameters for 3D‐MLC vs clinical GRID‐block plans using an upper bound of *P* < 0.05.

### 2.7 Simulating dose escalated plans

It has been reported in the GRID therapy literature that therapeutic gain can be achieved by escalating dose > 12 Gy in one fraction.^8^, ^16^, ^22^, ^27^ However, for deep‐seated bulky tumors, a single‐field Cerrobend GRID‐block could potentially produce difficulties in avoiding skin toxicity. Therefore, our current clinical practice for GRID treatment is limited to 15 Gy (maximum point dose) in one fraction. Using the 3D‐MLC crossfire technique, we have simulated plans in Eclipse (for all patients) with dose escalation schemata of 15 to 23 Gy as a function of skin dose. As mentioned above, RTOG‐0915 single‐fraction dose limits were used to evaluate the dose‐escalated GRID plans.

## 3 RESULTS

**Figure 2:**
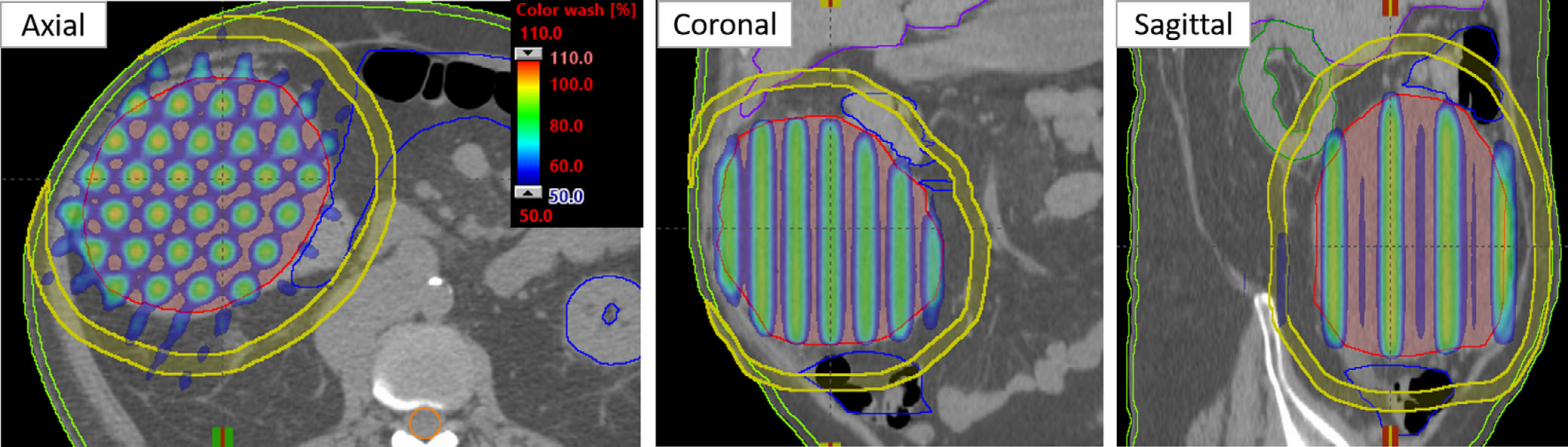
The isodose colorwash in the axial, coronal and sagittal views is shown for a three‐dimensional‐multileaf collimator (3D‐MLC) GRID plan of example patient #12. The original GTV size was 15 cm (in diameter) in the right abdomen. The prescription was 15 Gy in 1 fraction, allowing maximum point dose of 110% at the tumor center. Utilizing the 3D‐MLC cross‐fire technique, skin was spared dramatically (see all three views) while also respecting dose tolerances of the other internal structures such as large bowel (blue), liver (purple) and right kidney (dark green). Yellow color ring was contoured to calculate D2cm (%) for GRID target.

All 3D‐MLC crossfire GRID plans exhibited excellent target coverage with a mean GTV dose of 13.5 ± 0.5 Gy (range: 12.43–14.24 Gy) and a mean PVDR of 2.0 ± 0.3 (range: 1.7–2.4). The average values of maximal dose and dose to 5 cc of skin were 9.9 ± 3.1 Gy (range: 5.4–14.0 Gy) and 6.2 ± 1.9 Gy (range: 4.1–11.1 Gy) respectively. An example isodose colorwash in axial, coronal, and sagittal views is shown in Fig. 2. In this case, the internal critical structures such as large bowel, liver, and right kidney were also spared in addition to skin dose tolerances while using the 3D‐MLC crossfire technique. In Table 2, the skin to tumor center distance is shown for all patients. Additionally, D2cm from the GRID GTV and the maximal dose to immediately adjacent critical structures were documented. In this patient cohort, the distance from skin to tumor center ranged from 4.3 to 10.4 cm. The D2cm ranged from 55% to 75% depending upon tumor size. Critical organ dose tolerances were respected per RTOG requirements for immediately adjacent critical structures (spinal cord, ribs, bowel, and stomach; see Table 2).

**Table 2:** Distance to tumor center, D2cm, and dose to critical organs adjacent to the Gross tumor volume from the three‐dimensional‐multileaf collimator (3D‐MLC) plan for the patients included in this study.

Pt. #	Treatment site	Distance from skin to tumor center (cm)	D2cm (%)	Maximal dose to immediately adjacent critical structures (Gy)
1	Left lung	9.9	70.9	6.7 (spinal cord)
2	Left neck	4.3	61.1	5.9 (spinal cord)
3	Right axilla	7.5	64.6	7.3 (ribs)
4	Left kidney	10.4	72.1	6.4 (bowel)
5	Right neck	6.2	63.6	8.0 (spinal cord)
6	Right kidney	9.9	75.1	5.6 (spinal cord)
7	Thyroid	5.0	55.2	6.8 (spinal cord)
8	Chest	7.0	71.1	5.6 (spinal cord)
9	Chest	6.9	62.6	5.9 (spinal cord)
10	Abdomen	8.0	73.6	5.7 (stomach)
11	Liver	5.9	70.1	5.6 (spinal cord)
12	Right adrenal	8.7	71.3	7.9 (large bowel)
13	Right thigh	6.6	72.1	6.7 (bowel)

In the physics second check, the independent MU calculation (using an in‐house TMR‐based spreadsheet method) agreed within ±3.0%, on average, of the 3D‐MLC plans. Additionally, the in‐house MC algorithm showed that the planned dose agreed within ±2.0% of the MC computed dose.

**Figure 3:**
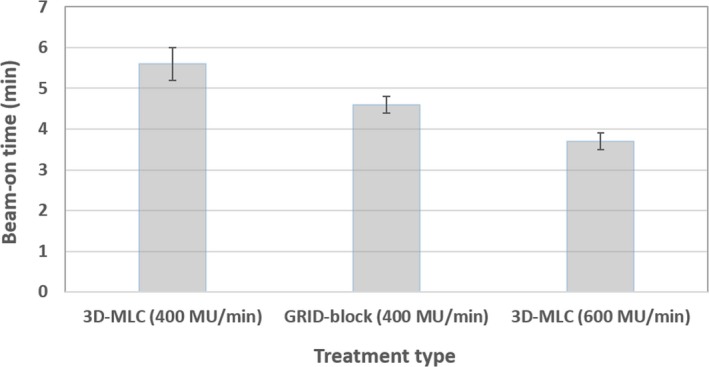
The beam‐on time for GRID‐block vs three‐dimensional‐multileaf collimator (3D‐MLC) plans for all 13 patients. Mean values of beam‐on time for GRID‐block and 3D‐MLC plans were 4.6 ± 0.2 min (ranged, 4.33–4.94 min), 5.6 ± 0.4 min (ranged, 5.0–6.2 min) with 400 MU/min and 3.7 ± 0.2 min (ranged, 3.33–4.16 min) while re‐calculating 3D‐MLC plans with 600 MU/min, respectively; with 3D‐MLC plans consistently improving the beam‐on time. However, due to gantry rotation time in the 3D‐MLC plans the overall treatment time would be similar.

The comparison of beam‐on time for 3D‐MLC vs clinical GRID‐block plans for all 13 patients is shown in Fig. 3. The 3D‐MLC grid plans provided 420 ± 138 (range: 183–606) (*P* < 0.001) higher MU than the clinical single‐field GRID‐block plans. For a fair comparison, 3D‐MLC plans were first calculated with a dose rate of 400 MU/min, identical dose rate to clinical cerrobend GRID‐block plans. Mean values of beam‐on time for the GRID‐block and 3D‐MLC plans were 4.6 ± 0.2 min (ranged, 4.33–4.94 min) and 5.6 ± 0.4 min (ranged, 5.0–6.2 min) respectively. However, while utilizing the 600 MU/min dose rate for 3D‐MLC plans, the resulting beam on time was about 0.8 ± 0.3 min (range: 0.4–1.3 min) (*P* < 0.001) less, on average, compared to traditional GRID‐block plans. Therefore, the data suggest that overall treatment times (while accounting for gantry angles rotation time for 3D‐MLC plans) would be similar between the plans.

**Figure 4:**
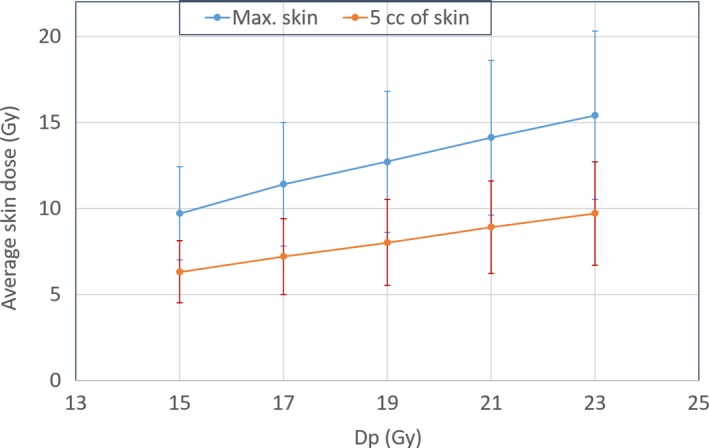
Calculation of predicted average skin doses (maximal and dose to 5 cc of skin) as a function of escalated prescription doses (Dp) for all 13 GRID therapy patients. A simple three‐dimensional‐multileaf collimator crossfire GRID planning technique allowed for escalation of tumor doses up to 23 Gy while maintaining the skin toxicity.

Using all 13 patients listed above, the predicted skin mean and standard deviation values of skin doses as a function of escalated prescription dose (Dp) for all simulated 3D‐MLC crossfire plans is shown in Fig. 4. RTOG‐0915 guidelines (single fraction, Arm 1) for OAR dose limits were followed. It was observed that with 3D‐MLC plans, tumor doses could be escalated up to 23 Gy while respecting skin dose tolerances. Furthermore, other internal OAR dose tolerances were also under the requirement set by RTOG guidelines.

## 4 DISCUSSION

In this report, we have presented a novel 3D‐MLC crossfire treatment planning technique and clinical implementation of treatment delivery for a single high‐dose GRID therapy treatment (15 to 23 Gy) to deep‐seated bulky tumors. For a maximum prescription dose of 15 Gy, a mean GTV dose of >13.5 Gy, on average, was achieved for the deep‐seated tumors while significantly sparing the skin and other internal critical structures. An average PVDR of 2.0 was achieved, but can be varied by changing the GRID lattice distance as needed. Our PVDR results were consistent with a glioblastoma case study reported by Jin et al.^13^ However, in their study the GRID therapy plan was inversely‐optimized with a simultaneous integrated boost (SIB) for many spheres generated inside the target. Therefore, the plan needed additional treatment planning and optimization time as well as patient‐specific QA due to MLC modulation. Also, there was no reported total number of MU and beam on time in this case. In contrast, we have used a simple, yet clinically useful 3D‐conformal forward planning approach that does not require patient‐specific QA (due to no beam modulation) and can be delivered within a few minutes.

With this technique, for extracranial bulky deep‐seated tumors, there are 6 co‐planar gantry angles available that provided special tunneling directions with 90‐degree collimator rotation. Comparable total number of MU (compared to traditional GRID‐block) and a relatively shorter beam‐on time with the 3D‐MLC crossfire technique is clinically appealing in the management of the deep‐seated bulky lesions. Additionally, our simulation study suggests that tumor dose can be escalated up to 23 Gy with 3D‐MLC crossfire technique while avoiding skin toxicity. Although, treatment efficacy of escalating higher doses for tumor control and treatment related toxicities needs prospective clinical follow‐up of GRID therapy patients.

A major difference of our study from the previous two‐dimensional‐GRID therapy approach,^1^, ^2^, ^3^, ^16^, ^22^ tomotherapy or MLC‐based studies^4^, ^5^, ^6^, ^7^, ^8^, ^9^, ^10^, ^11^, ^12^, ^13^, ^14^, ^15^ was that our treatment planning approach uses an MLC‐based, 3D‐conformal forward planning technique with no beam modulation. Therefore, this MLC cross‐firing procedure preserves the characteristics of 3D‐conformal radiation therapy and provides all dosimetry information without the need for patient‐specific QA. Clinical and biological data suggest that the success of GRID therapy in shrinking large tumors depends on the high PVDR. One potential concern is the dose blurring due to tumor motion in GRID therapy.^26^ Even with relatively shorter beam on times, but similar overall treatment time compared to traditional GRID‐block, our 3D‐MLC crossfire plans could potentially be delivered using image‐guidance procedure. Furthermore, this time can be reduced by using recently adopted flattening filter free (FFF) beams^31^, ^32^ for 3D‐MLC plans. With the use of FFF‐beam for 3D planning, the instantaneous dose rate has increased by approximately a factor of 2.33 with 6X‐FFF beam (1400 MU/min). It could increase up to by a factor of 4 with 10X‐FFF beam (2400 MU/min). Therefore, due to much shorter beam on time with 3D‐MLC crossfire technique, deep inspiration breath‐hold (DIBH) GRID therapy planning with FFF‐beams may be of value in future investigations.

In summary, the potential benefit of a simple yet clinically applicable treatment planning and delivery approach using 3D‐MLC crossfire was proposed for GRID therapy patients with deep‐seated bulky tumors. Each 3D‐MLC plan was rigorously evaluated using the single‐dose RTOG dosimetric compliance criteria for OAR dose tolerances. With 3D‐MLCs, faster and more effective treatment delivery is possible, with the potential benefit of tumor dose escalation, if desired. Additionally, 3D‐MLC plans overcome concerns of the accuracy of the dose calculation and delivery errors for small fields (beamlets) while using step‐ and shoot‐IMRT MLC‐modulation or tomotherapy delivery. Moreover, the 3D‐MLC forward planning method eliminates patient‐specific IMRT quality assurance, thus potentially offering cost‐effective same day GRID therapy treatments. Since MLCs are integral parts of each medical linear accelerator (by now), our technique can be easily adopted to other small radiotherapy clinics with less extensive physics or machine support for GRID therapy patients. Our future work includes the following: generating an MLC‐based GRID template in Eclipse for automation, prospectively quantifying the therapeutic gain and treatment related toxicity^8^, ^16^, ^22^, ^27^ by escalating tumor dose to the deep‐seated tumors and potentially using DIBH with FFF‐beams^31^, ^32^ in the management of tumor motion for the MLC‐based GRID therapy patients. Moreover, the potential use of the 3D‐MLC crossfire approach for highly irregular GRID targets will be explored.

## 5 CONCLUSIONS

A simple yet clinically useful 3D‐conformal MLC‐based crossfire GRID‐therapy technique resulted in enhanced target coverage for the deep‐seated bulky tumors with reduced skin toxicity and other internal critical structures. This simple MLC‐based approach can be easily adopted by any radiotherapy clinic. It provides detailed dosimetry and a safe, effective treatment modality by eliminating the heavy physical GRID‐block without beam modulation. Moreover, using the 3D‐MLC approach, our simulation study suggests that tumor dose can be escalated up to 23 Gy while avoiding skin toxicity. A prospective clinical trial is underway to evaluate the tumor local control rates and treatment related toxicity with an escalated‐dose for patients with 3D‐MLC GRID therapy.

## CONFLICT OF INTEREST

No conflict of interest.
